# Quality of survey-based study reports in dentistry

**DOI:** 10.1186/s12903-023-02979-z

**Published:** 2023-05-23

**Authors:** Manuel Antonio Mattos-Vela, Teresa Angélica Evaristo-Chiyong, Kariem Siquero-Vera

**Affiliations:** Facultad de Odontología, Grupo de investigación SAETA, Universidad Nacional Mayor de San Marcos, Calle Germán Amézaga 375. Lima 1, Lima, Peru

**Keywords:** Surveys and questionnaires, Dental health surveys, Epidemiologic studies, Health surveys, Dentistry

## Abstract

**Background:**

Surveys are a widely used research method in dentistry in different specialities. The study aimed to determine the quality of survey-based research reports published in dentistry journals from 2015 to 2019.

**Methods:**

A cross-sectional descriptive research study was conducted. The report quality assessment was carried out through the SURGE guideline modified by Turk et al. Four journals indexed in the Web of Science were selected: BMC Oral Health, American Journal of Orthodontics and Dentofacial Orthopedics, Journal of Dental Education, and Journal of Applied Oral Science. The selection of articles was made using the PubMed database considering the following search words: questionnaire OR survey, two trained reviewers applied the guideline to the selected articles, and the controversies were solved by discussion and consensus.

**Results:**

A total of 881 articles were identified, of which 99 met the selection criteria and were included in the study. The best-reported items (n = 99) were four: the two that described the introduction of a study, the results reflecting and concerning the study objectives, and the review by an ethics committee. Five items were poorly reported: to declare the incentives to study participants (n = 93), three items on the description of statistical analyses (n = 99, 99, and 94), and information on how nonrespondents differed from respondents (n = 92).

**Conclusions:**

There is a moderate quality of reporting of all aspects that should be considered in survey-based studies in dentistry journals. Poorly reported criteria were found mainly in the statistical analysis.

## Background

Surveys are a widely used research method in dentistry in its different specialities, but mostly in public health, ethics, and education [1, 2]. This method can be applied in quantitative, qualitative, or mixed research; it allows for collecting information on a specific topic through low-cost questionnaires that are easy to apply [3, 4].

Research studies using surveys are as important as any other type of research; they are the beginning of exploratory studies, as well as cross-sectional axes in quantitative research. They are the basis for going on to the next levels of evidence, thus allowing for a comprehensive approach to health research, being used to address issues that are difficult to evaluate and allowing the generation of constructs in a specific topic [5, 6].

In a study conducted by Bennett et al. [5] on the evaluation of the quality of survey reports in the medical field, in 117 published studies, it was found that several criteria were poorly reported: few studies provided the survey or core questions (35%), reported the validity or reliability of the instrument (19%), defined the response rate (25%), discussed the representativeness of the sample (11%) or identified how they handled missing data (11%). Other studies evaluating the quality of survey-based study reports found similar results, e.g., Turk et al. [6] in different medical disciplines, Li et al. [7] in the area of nephrology, Pagano et al. [8] in the area of transfusion medicine, and Rybakov et al. [9] in the area of pharmacy. However, no studies were found evaluating the quality of survey reports in dentistry.

Science grows with the production of scientific knowledge informed through research articles, which should allow for evaluating the quality of the study conducted. It is necessary to know how much of the survey-based dentistry research published in high- and medium-impact journals is useful and has been clearly and completely reported. It is necessary to identify where it is failing and what needs to be improved so that these reports are useful for the profession, systematic reviews, and science. The study aimed to determine the quality of survey-based research reports published in dentistry journals from 2015 to 2019.

## Methods

### Type of study

A descriptive, cross-sectional investigation was carried out.

The study was approved at the institutional level, and evaluation by a research ethics committee was not considered necessary because it was a documentary evaluation that did not include human subjects.

### Population and sample

Articles published from 2015 to 2019 in four dentistry journals indexed on the Web of Science. The following journals were selected: BMC Oral Health, American Journal of Orthodontics and Dentofacial Orthopedics, Journal of Dental Education, and Journal of Applied Oral Science because they are high- and medium-impact journals (Q1 and Q2) that mostly publish survey-based articles, which was determined by reviewing a pilot sample of 140 articles published in dentistry journals during 2019, found in the PubMed database and based on surveys. A 5-year time period was considered for the search for articles based on previous studies that used a variable time range, between 1 and 17 years [6, 8, 9].

#### Inclusion criteria

original survey studies that used a self-administered questionnaire as the primary research instrument (to answer its primary objective), cross-sectional surveys, and studies published in English.

#### Exclusion criteria

studies for the validation of an instrument that examined only the psychometric characteristics of the instrument, surveys administered through the web (online), study designs (randomized clinical trials, cohort studies, and case-control studies) where surveys were only used for demographic data, other types of studies (reviews, letters, commentaries, etc. ), studies that were part of a larger investigation, studies that performed a secondary analysis of the survey, studies that used semistructured interviews instead of questionnaires and surveys sent by e-mail.

### Article selection procedure

This study defines a survey as the research method by which information is collected by asking people written questions about a specific topic, and the data collection procedure is standardized and well-defined [3].

The PubMed database was used to select the articles for each of the selected journals using the following search words: questionnaire OR survey, filtering by publication date from January 1, 2015, to December 31, 2019.

Two reviewers independently selected all references (title and abstract) and excluded those that did not meet the established criteria. In the second stage, the full-text articles were reviewed to determine which would be included in the study. In both stages, after the independent review, the researchers compared their results, and in cases of discrepancy, these were discussed and agreed upon.

### Criteria for evaluating the quality of the report

The quality of the articles was evaluated independently and in duplicate by two other reviewers. Disputes were resolved by discussion and consensus.

To evaluate the quality of the research reports, the SURGE instrument modified by Turk et al. [6] was chosen, containing 33 items, of which only one item was modified, and the telephone survey mode, which was eliminated since only self-administered questionnaires were evaluated. This instrument was tested by two researchers on a convenience sample of survey items identified by the authors. No modifications had to be made to the content or wording.

The following variables were also recorded to characterize the sample: year of publication, continent of origin and journal.

### Pilot study

A pilot study was conducted to train and standardize criteria for the search and selection of the articles, according to the established selection criteria, as well as for the application of the quality criteria of the report.

### Statistical analysis

Data processing and analysis were carried out using the statistical program SPSS v 26 (SPSS Inc., IL, USA). Descriptive statistics were applied to the study variables using frequency distribution tables.

## Results

**Fig. 1:**
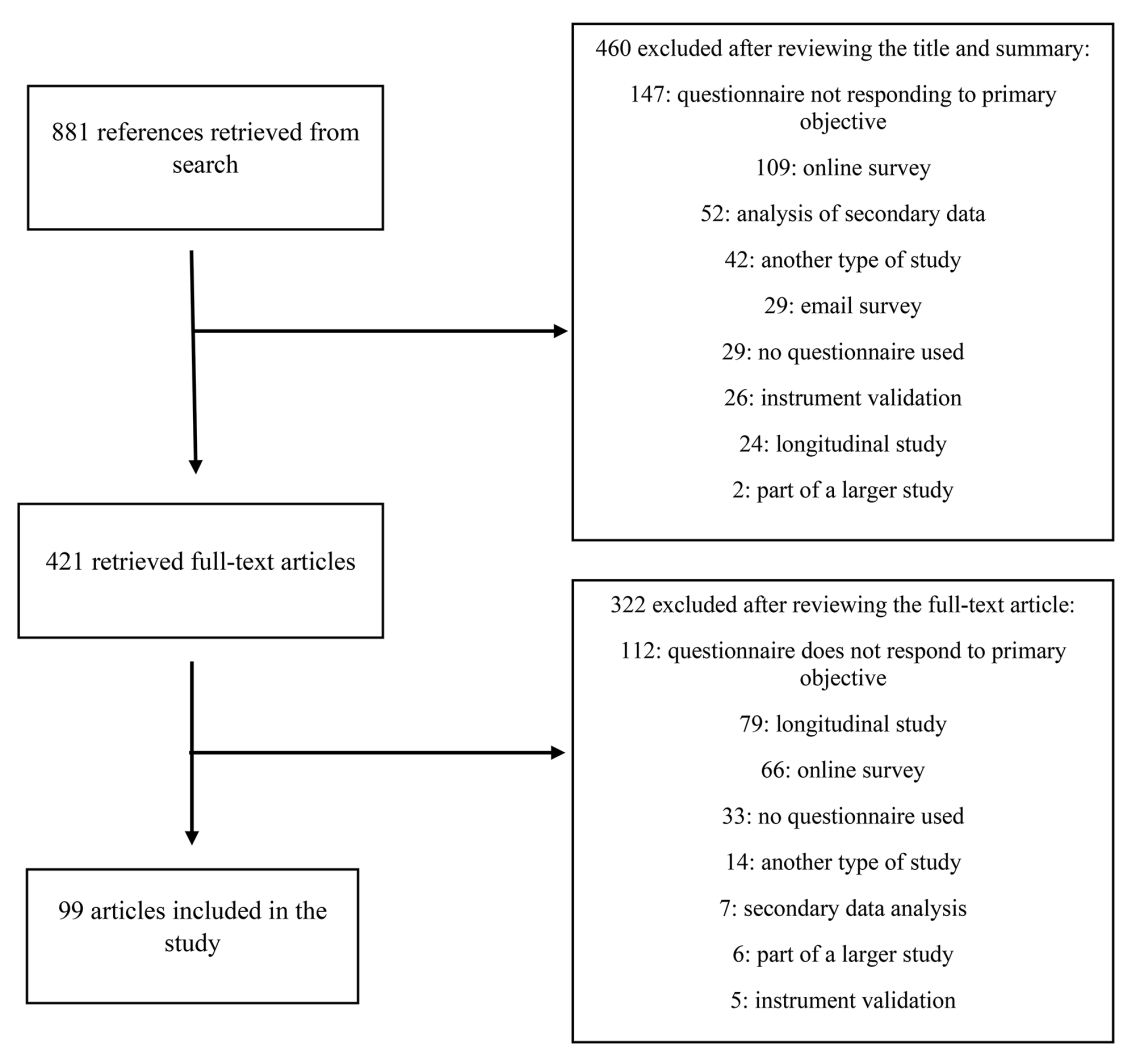
Flowchart of selected articles for the study

From the bibliographic search, 881 references were retrieved; then, their titles and abstracts were read, and 460 were excluded according to the established criteria. From the other references, the full text of the articles (n = 421) was obtained and evaluated to determine compliance with the selection criteria, excluding 322 articles and considering only 99 in the study to apply the SURGE guidelines and evaluate the quality of the report (Fig. 1). In the study sample, the most frequent articles were those published in 2018 (n = 29), those from the Americas (n = 41), and those published in the journal BMC Oral Health (n = 43) (Table 1). The percentage value is not mentioned in all results because it matches the absolute value.

**Table 1:** Characteristics of the articles evaluated

Variables		n
Year of publication		
	2015	17
	2016	15
	2017	20
	2018	29
	2019	18
Continent of origin		
	America	41
	Europe	18
	Africa	6
	Asia	29
	Oceania	5
Journal		
	BMC Oral Health	43
	AJODO	13
	JDE	41
	JAOS	2

Table 2 shows the evaluation results of the title, abstract, introduction, and methods of the articles through 21 criteria of the SURGE instrument.

**Table 2:** Evaluation of articles according to SURGE criteria: title, abstract, introduction, and methods

Section	Description of the criterion	Categories	n
Title and abstract	It states the word ‘questionnaire’ or ‘survey’ in the title and/or abstract.	1. In title and abstract	19
		2. In title or abstract	78
		3. No	2
Introduction	There is an explanation of why the research is necessary.	1. Yes	99
		2. No	0
	It indicates the purpose or objective	1. Yes	99
		2. No	0
Methods	It describes the questionnaire/provides access to the items in the questionnaire	1. Questionnaire provided	45
Research instrument		2. Central questions provided	26
		3. A complete question provided	0
		4. Questions not provided	28
	If an existing instrument was used, its psychometric properties were mentioned: confidence and validity	1. Yes	18
		2. No	32
		3. Not applicable	49
	If an existing instrument was used, references to the original study are provided.	1. Yes	47
		2. No	3
		3. Not applicable	49
	In a new instrument, the procedures used to develop it and/or the methods used for the pretest are mentioned.	1. Yes	26
		2. No	23
		3. Not applicable	50
	In a new instrument, its validity and confidence were reported.	1. Both	4
		2. Only confidence	7
		3. Only validity	2
		4. None	36
		5. Not applicable	50
	It describes the scoring procedures	1. Yes	77
		2. No	6
		3. Not applicable	16
Sample selection	It describes the study population and sampling frame.	1. Both	46
		2. Study population	28
		3. Sampling frame	1
		4. None	24
	It describes the representativeness of the sample.	1. Yes	87
		2. No	12
	It presents a sample size calculation or justification of it.	1. Yes	81
		2. No	18
Survey administration	The mode of administration of the survey to participants is specified.	1. In person	75
		2. Mail	6
		3. Mixed	5
		4. Not mentioned	13
	Type and number of contacts	1. Type and number	33
		2. Only type	53
		3. No information	13
	Financial or other incentives to study participants.	1. Yes	6
		2. No	93
	Description of who approached potential participants.	1. Yes	38
		2. No	61
Analysis	It describes the method of data analysis.	1. Adequate (complete)	91
		2. Inadequate (incomplete)	4
		3. Does not describe	4
	It mentions the methods for nonresponse error analysis.	1. Yes	0
		2. No	99
	It mentions the method for calculating the response rate.	1. Yes	0
		2. No	99
	It mentions definitions for complete versus partial endings.	1. Yes	37
		2. No	62
	It mentions methods for handling missing item data.	1. Yes	5
		2. No	94

It was found that most articles used the term *survey* or *questionnaire* in the title or abstract (n = 97). All articles explained why the research was necessary and indicated the study objective.

In the section on methods, almost half of the articles provided the questionnaire (n = 45) and used an existing instrument (n = 50). In this case, 32 did not mention their psychometric properties, and three did not provide references to the original work. Among the papers that used a new instrument (n = 49), 23 did not mention the procedures used to develop it or the methods used for the pretest, while 36 did not report the validity and confidence of the instrument. Among the studies that used a questionnaire that required scoring (n = 83), six did not describe the scoring procedures.

Regarding the evaluation of the sample selection, few studies did not describe the study population and the sample framework (n = 24), the representativeness of the sample (n = 12), the calculation of the sample size, or the justification thereof (n = 18).

Regarding survey administration, few articles mentioned the mode of administration or the type and number of contacts (n = 13); however, most of them did not report the incentives to respondents (n = 93) or who approached potential participants (n = 61).

In the evaluation of the statistical analysis, four articles did not describe the method of analysis, while none reported the methods for the analysis of the nonresponse error and the calculation of the response rate; most failed to mention definitions for complete versus partial endings (n = 62) and methods for handling missing data (n = 94).

Table 3 describes the evaluation of the results, discussion, and ethical aspects of the articles through 12 criteria of the SURGE instrument.

**Table 3:** Article evaluation according to SURGE criteria: Results, discussion, and ethics

Section	Description of the criterion	Categories	n
Results	It reports the response rate.	1. Yes, defined	57
		2. Yes, not defined	14
		3. Partial information	4
		4. No information	24
	It takes into account all respondents.	1. Yes	86
		2. No	13
	Information on how nonrespondents differ from respondents.	1. Yes	5
		2. Subject addressed	2
		3. No information	92
	Results are clearly presented.	1. Yes, complete	97
		2. Yes, partial	2
		3. No	0
	Results reflect the objectives of the study.	1. Yes	99
		2. No	0
Discussion	Summary results in relation to the objectives of the study.	1. Yes	99
		2. No	0
	It mentions the strengths of the study.	1. Yes	53
		2. No	46
	It indicates the limitations of the study.	1. Yes	90
		2. No	9
	There is an explicit discussion of the generalization of results.	1. Yes	65
		2. No	34
Ethical quality indicators	Report on the financing of the study.	1. Yes	42
		2. No	57
	Review of the study by a Research Ethics Committee.	1. Yes	86
		2. Reported exempted from a committee	13
		3. No	0
	Report on procedures for individuals’ consent	1. Yes	69
		2. Reported refused informed consent	3
		3. No	27

In the results section, 24 articles did not report the response rate, 13 did not consider all respondents, and 92 did not report the difference between respondents and nonrespondents. However, in all the articles, the results were presented clearly and in relation to the study objectives.

For the Discussion section, only one criterion was correctly reported in all cases, and the results were summarized in relation to the study objectives, while 46 and six articles did not mention the strengths and limitations of the study, respectively. In addition, 34 studies discussed the generalization of results.

Finally, regarding ethical quality indicators, all articles reported on the review of the study by an ethics committee, while 57 and 27 articles did not report on the funding and procedures of respondent consent, respectively.

## Discussion

In the evaluation of the quality of survey-based studies, it was found that the best-reported sections were title and abstract, introduction, sample selection, results, and discussion; specifically, there were 15 criteria very well reported (with a frequency greater than 80%), most of them within the aforementioned sections. Bennett et al. [5], Pagano et al. [8], and Rybakov et al. [9] also found the title and abstract, introduction, and discussion sections well reported. It is possible that the STROBE [10] guidelines, which are the guidelines for good reporting of observational studies required by health science journals, may have contributed to this. In addition, the recommendations given for writing the items in these sections are well-known and easy to comply with.

There are 12 criteria where most articles performed a bad report, and five of them were poorly reported (with a frequency greater than 80%): incentives to study participants; mentioning methods for nonresponse error analysis, calculating the response rate, and handling missing item data; and reporting how nonrespondents differed from respondents. Three of these criteria belong to the analysis section. Other investigations found a greater number of misreported criteria [5, 6, 8]; one even observed as many as 21 inadequately reported items in medical articles [5]. The items mentioned methods for nonresponse error analysis [5, 6, 8, 9] and for handling missing item data [5, 8, 9] were also poorly reported in other research studies.

This research allows warning about the aspects that should be improved in the reporting of studies based on self-administered surveys in the field of dentistry. In the Methods section, it should be emphasized that when the research is conducted using a new or existing questionnaire, the psychometric properties of the questionnaire should be mentioned. The vast majority of researchers in the studies evaluated considered it sufficient to mention only the reference to the original validation work when working with an existing questionnaire.

The SURGE guidelines [11], unlike STROBE [10], develop more precisely what needs to be reported in terms of the survey administration. This research has demonstrated that three out of the four items that should be described on this aspect, according to the SURGE criteria, were not performed correctly, which does not allow a study to be replicable or a reader or reviewer to assess its quality and the possible introduction of bias.

The statistical analysis description is a critical aspect in the communication of this research and it is necessary to train researchers in this field since statistical knowledge is an important element to prevent his or her study from lacking methodological validity. This study found that four out of the five items that SURGE recommends reporting in this area were poorly reported, which agrees with other investigations in which the quality of survey-based studies in areas such as general medicine [5, 6], transfusion medicine [8], and pharmacy [9] were evaluated. A previous study noted the poor reporting of statistical aspects in articles of different research designs in dentistry journals [12]. Although it is common for articles to report the value of response rate, no one mentioned the method for calculating it; SURGE asks to mention both in the results and methods sections, respectively.

It is also important to note that the description of how nonrespondents differ from respondents should be improved in the results section. It may be infrequent to mention this aspect because of the additional work it would take researchers to obtain this information, as it can be difficult to obtain because of the lack of access to the group of non-respondents. However, where possible, the researcher should report it, which will help to make transparent the representativeness of the sample studied in relation to the population.

Regarding the ethical aspects of a research study, improved reporting of the study’s financing is necessary. It would be advisable for journals to require authors to submit their manuscripts with this information. For example, one of the journals evaluated in this study, BMC Oral Health, provided the communication of ethical aspects since, at the end of the article, they presented sections where the authors had to declare the financing of the study, the approval of an ethics committee, and the consent of participants.

As far as the authors are aware, this is the first study that evaluates the quality of survey-based research in the area of dentistry, which warns of the aspects that should be improved for clearer, more complete, and transparent communication of this type of study, considering that the use of surveys in research in the area of health sciences is frequent [1].

One limitation of this study was that the evaluation of some quality items was not simple, and the agreement reached by the two evaluators of the article could be different from what was interpreted and evaluated in other studies that also used the SURGE criteria [5, 6, 8, 9]. For example, in some of the items evaluated, the report was considered valid even though the information was not found in the corresponding section; it was sufficient that it was present somewhere in the article. Moreover, several articles evaluated used nonprobabilistic samples, so the sampling frame was not reported since it was unnecessary. In these cases, this item was not considered misreported. There is no extended version of SURGE where the criteria for evaluating each item are explained in detail, as there is for the STROBE [13] and CONSORT [14] statements, among others.

The results of this study are not necessarily generalizable to articles published in all dentistry journals since it only evaluated four journals; however, it is the first report that provides evidence in this field.

## Conclusions

It is concluded that there is a moderate quality of reporting of all the aspects to be considered for studies based on self-administered surveys in four dentistry journals. Poorly reported criteria were found mainly in the statistical analysis section.

## Data Availability

The datasets generated and analysed during the current study are available in the Zenodo repository, 10.5281/zenodo.7391366.
